# Long COVID and Reduced Thrombosis in Antihistamine-Treated Patients: An Observational Study in the Metropolitan Area of Barcelona

**DOI:** 10.3390/v18020197

**Published:** 2026-02-02

**Authors:** Anna Puigdellívol-Sánchez, Antonio Arévalo-Genicio, Mª Carmen García-Arqué, Marta Gragea-Nocete, Celia Lozano-Paz, Vanessa Moro-Casasola, Cristina Pérez-Díaz, Roger Valls-Foix, Ramon Roca-Puig, Maria Llistosella

**Affiliations:** 1 Primary Health Care, CAP Anton de Borja-Centre Universitari, Consorci Sanitari de Terrassa (CST), c/Marconi-Cantonada Edison s/n, 08191 Rubí, Spain; 2 Primary Health Care, CAP Dr. Joan Planas, Consorci Sanitari de Terrassa (CST), Av. Pau Casals, 12, 08755 Castellbisbal, Spain; 3 Human Anatomy and Embryology Unit, Faculty of Medicine, Universitat de Barcelona, c/Casanova 143, 08036 Barcelona, Spain; 4 Primary Health Care, CAP St. Genís (CST), Carrer Miquel Mumany 11, 08191 Rubí, Spain; 5 Primary Health Care, CAP St. Llàtzer–Centre Universitari (CST), c/de la Riba 62, 08221 Terrassa, Spain; 6 Research and Innovation, Hospital de Terrasa-Hospital Universitari (CST), Carretera de Torrebonica s/n, 08227 Terrassa, Spain; 7 Primary Health Care, CAP Can Roca (CST), c/Fàtima 18, 08225 Terrassa, Spain

**Keywords:** long COVID, thrombosis, vaccine, COVID-19

## Abstract

Background: Early evidence from a nursing home in Yepes (Toledo, Spain) indicated that antihistamines combined with azithromycin prevented deaths and hospitalizations during the first COVID-19 wave. Subsequent data from the Consorci Sanitari de Terrassa (CST) showed that patients chronically taking antihistamines had significantly reduced hospital admissions and mortality. However, a concerning rise in long COVID incidence (2–5%) after the third infection and a doubling of thrombosis rates in patients over 60 were observed. Objective: This study aimed to determine whether chronic antihistamine prescription is associated with a reduction in long COVID syndrome and thrombotic events. Methods: We analyzed anonymized data from the CST population (n = 192,651 as of March 2025). Variables included age, gender, chronic antihistamine use, number of chronic treatments (nT), COVID-19 vaccination status, SARS-CoV-2 infection history, long COVID (LC) incidence, and aggregated thrombotic events. Odds ratios (OR) were calculated using chi-square tests. Results: The prevalence of LC increased progressively with successive infections in the non-antihistamine group. No significant differences were found with the antihistamine group, which presented no LC cases among the 52 patients with three documented infections. Thrombotic events were significantly less frequent in antihistamine users with at least one chronic prescription (*p* < 0.0001). Conclusions: Results suggest a protective effect of antihistamines against thrombotic events. While confirmation via multicenter, randomized trials is needed, a pragmatic approach using antihistamines could be considered for symptomatic patients in the early stage of infection.

## 1. Introduction

SARS-CoV-2 infection is associated with long COVID syndrome [1] and other long-lasting multi-organ effects [2,3], including cardiovascular sequelae [4].

Long COVID has been defined as ‘the continuation or development of new symptoms 3 months after the initial SARS-CoV-2 infection, with these symptoms lasting for at least 2 months with no other explanation’ [5]. After matching anonymized population-based data from more than 190,000 patients, including over 40,000 infection records, with survey responses and specific differential diagnostic tests performed in hundreds of symptomatic respondents, the prevalence of long COVID was shown to increase substantially following successive infections. Furthermore, overall thrombotic events, including ischemic cardiopathy, ischemic stroke, pulmonary thromboembolism, and thrombosis of retinal vessels, doubled from 2020 to 2024 in patients over 60 years old, even in those not on chronic treatments [6]. The increasing incidence of thrombotic events following COVID-19 infection has been described since the early stages of the pandemic. Thrombotic events may occur from as early as two weeks [7] or up to 49 weeks post-COVID [8]; however, to date, no specific post-infection treatments have been protocolized, and no conclusive evidence exists regarding the use of specific antiplatelet therapies [9].

At present, nirmatrelvir remains the only approved therapeutic for the early treatment of COVID-19 in elderly patients, having demonstrated efficacy in reducing hospitalizations, intensive care unit (ICU) admissions, and mortality among high-risk individuals [10]. Nonetheless, evidence indicates that it neither prevents infection among close contacts [11] nor reduces progression to long COVID [12]. Additional barriers to its widespread adoption include its substantial cost and a significant potential for drug–drug interactions.

During the early stages of the pandemic, several targets were explored for drug repurposing. In one study, 29 FDA-approved drugs were identified as potential inhibitors of the interaction between host factors and the SARS-CoV-2 virus [13], including the antihistamine loratadine. Preliminary evidence of a protective effect from pragmatic use emerged from an experience at the Yepes nursing home for the elderly (Toledo, Spain). During the first wave, all residents became infected, but none died or required hospital admission after receiving treatment with antihistamines and azithromycin [14]; this contrasted with a mortality rate of 40% in the surrounding area [15]. The use of the same treatment in primary care patients in the same area was associated with a reduction in hospital admissions compared to national rates in Spain [16]. A similar, significant reduction in hospital admissions and death was observed among patients on chronic antihistamine therapy in our large integrated health care consortium in the metropolitan area of Barcelona encompassing over 1400 hospital admissions from a population of over 150,000 patients [17].

The aim of the present study was to assess whether chronic antihistamine use was associated with a different risk of progression to long COVID syndrome and the development of thrombotic events following COVID-19.

## 2. Materials and Methods

The descriptive study of COVID-19 hospital admissions received approval from the Ethics Committee of the Consorci Sanitari de Terrassa (CST) on 8 April 2020 (ref. 02-20-161-021) and was registered as an observational trial at ClinicalTrials.gov on 29 April 2020 (NCT04367883). A subsequent amendment to analyze the variable of antihistamine treatment relative to the reference population was approved on 13 June 2022 (ref. 02-22-151-060) and posted on 17 August 2022 (NCT05504057; https://clinicaltrials.gov/study/NCT05504057, accessed on 30 November 2025). Finally, the study was expanded to include long COVID syndrome and thrombotic events on 24 February 2025. The planning, conduct, and reporting of the study were in accordance with the principles of the Declaration of Helsinki.

### 2.1. Socioeconomic Environment

The socioeconomic profile of the Consorci Sanitari de Terrassa (CST) has been described in previous publications [17,18]. In brief, the CST is a public healthcare provider for a population of 192,651 residents (as of March 2025) in Barcelona’s North Metropolitan Health Region. Its network of primary care centers serves a demographically diverse area comprising rural, residential, and metropolitan communities. Prior to the pandemic, life expectancy in the region consistently exceeded 81 years. Furthermore, COVID-19 vaccination coverage exceeded 90% among elderly patients with multiple chronic conditions, while infection rates were similar across all centers, ranging from 22% to 27%. The proportion of residents aged over 60 varies across centers from 15.1% to 24%, with most CST centers serving populations where over one-fifth of residents fall within this age group. The ‘Hospital Universitari de Terrassa’ serves as the reference hospital for these centers.

Further baseline details (gender, age, infection severity [hospitalization], comorbidity [number of chronic treatments], number of infections, and mean vaccine doses) are available in Supplementary Material File S0.

### 2.2. Quantification of Long COVID Syndrome Prevalence and Thrombosis

The Data Analysis Control Department collected anonymized data from the entire CST population without exclusions. This included information on COVID-19 infections, hospital admissions, long COVID syndrome (LC), and thrombotic events (Thr)—including strokes, myocardial infarction, pulmonary thromboembolism, and retinal vessel thrombosis—from March 2020 to March 2025. Data on gender, age, number of chronic treatments (nt), and COVID-19 vaccination status relative to the first infection (V preinf, V postinf, or NoV) were also collected. Patients receiving acute antihistamine treatment and no other chronic therapies were categorized as the AntiHm group (subgroup 0 nT). Case and hospitalization data have been analyzed previously [17,18]. The present study focuses specifically on the incidence of long COVID syndrome and thrombosis as long-term effects of COVID-19 infection.

The most likely SARS-CoV-2 variant responsible for each patient’s first infection was inferred by matching the infection date with the predominant variant circulating in Spain during that period (for being present in >60% of sequenced samples), using data from the Covariants website [19].

### 2.3. Statistical Analysis

Patients were stratified by gender, age (<60 or ≥60 years), number of SARS-CoV-2 infections, vaccination status, and number of chronic treatments (nT), as well as by the use of chronic antihistamine treatment (AntiHm or NoAntiHm). To calculate the odds ratio (OR) for the NoAntiHm/AntiHm comparison, only patients with at least one chronic prescription were included (≥1 nT), thereby excluding those with no chronic treatments. Subgroups were compared using chi-square tests via OpenEpi.com [20], applied only to categories containing at least five cases. A 95% confidence in interval (CI) for the OR has been calculated. Additionally, A Benjamini–Hochberg correction for multiple comparisons was applied. In the tables, significant comparisons surviving this correction are denoted by the ‘+’ symbol [21,22].

Complementary classic and Bayesian logistic regression analyses were performed using the JASP.org open-source statistical software [23] when the number of cases per category exceeded 10. For the dependent variable ‘long COVID’, covariates included nT (number of treatments), age, and number of SARS-CoV-2 infections; cofactors were gender, antihistamine use, and vaccination status prior to infection (V preinf). For the dependent variable ‘thrombosis’, the model also included vaccination status prior to thrombosis.

Collinearity between the covariates nT and age was assessed by calculating the Variance Inflation Factor (VIF). The VIF values were 1.598 and 1.584, respectively, which are well below the common threshold of 5, indicating no concerning multicollinearity [24].

## 3. Results

### 3.1. Long COVID Prevalence and Chronic Treatment with Antihistamines

The association between prevalent Long COVID (LC) and the number of infections, chronic treatments, vaccination status, and chronic antihistamine use is detailed in Table 1 and File S1. LC prevalence increased with the number of infections in the NoAntiHm group. Notably, no LC cases were identified among the AntiHm patients with two infections who had been vaccinated prior to their first infection, nor among the AntiHm patients with three or more infections. While no statistically significant differences were detected between the overall AntiHm and NoAntiHm groups, direct comparisons with specific polypharmacy subgroups were not possible, as most AntiHm subgroups either contained no cases or had fewer than five cases (see File S1).

### 3.2. Thrombotic Events in the COVID Era by Gender, Polypharmacy, Vaccination and COVID-19 Waves

**Figure 1:**
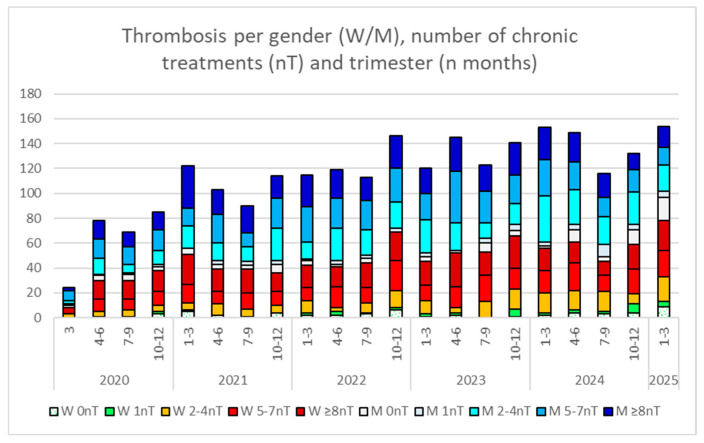
Total number of thrombotic events, stratified by gender (W, women; M, men), trimester (the specific number of months is indicated), and polypharmacy group (0, 1, 2–4, 5–7, or ≥8 number of chronic treatments [nT]), from the beginning of the pandemic in March 2020 (the first column represents only one month) to March 2025.

Up to 2458 thrombotic events were recorded between 1 March 2020, and 28 March 2025, and are presented in Figure 1. Additionally, 90 hemorrhagic ictus occurred that are not represented in the figure about thrombosis. A progressive rise in thrombosis is observed, with 429, 493, 529, and 550 cases from 2021 to 2024, respectively. Thrombosis appeared in all polypharmacy groups and also in patients with no chronic prescriptions to treat any comorbidity in both genders (see W 0 or M 0 in Figure 1). Significant gender differences were found (*p* < 0.0001), with 59.3% of the registered thrombosis occurring in men.

Despite diverse socioeconomic conditions (File S0), the incidence of thrombosis among patients aged >60 years was similar across most CST centers, ranging from 4.12% to 4.79% (File S4). A slightly lower rate was observed in one residential center (3.68%).

**Figure 2:**
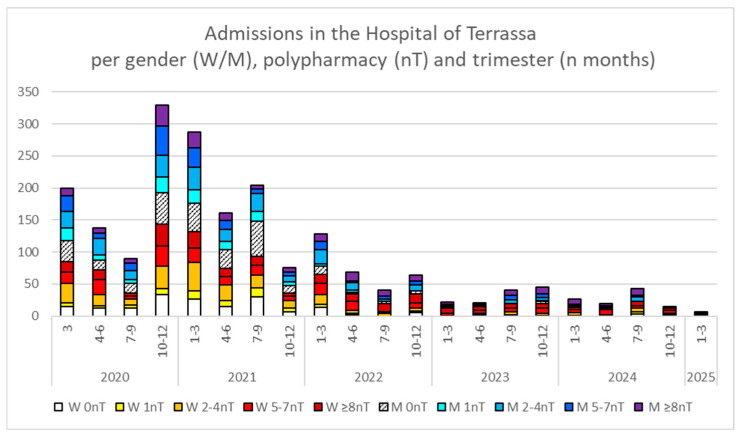
Total number of hospital admissions stratified by gender (W, women; M, men), trimester (the specific number of months is indicated), and polypharmacy group (0, 1, 2–4, 5–7, or ≥8 number of chronic treatments [nT]) from March 2020 to March 2025.

Although thrombotic events appeared more frequent during colder months (October to March in each season), no clear relationship was observed between thrombotic events and COVID-19 waves when comparing them with COVID-19 admissions at the institution’s reference hospital (Figure 2). Therefore, COVID-19 waves that peaked in summer—such as waves 7–9 in 2021 (the Delta wave) and those in 2023 and 2024, which included several Omicron waves that rose during July of those years—were not directly correlated with thrombotic events in the same trimester.

**Figure 3:**
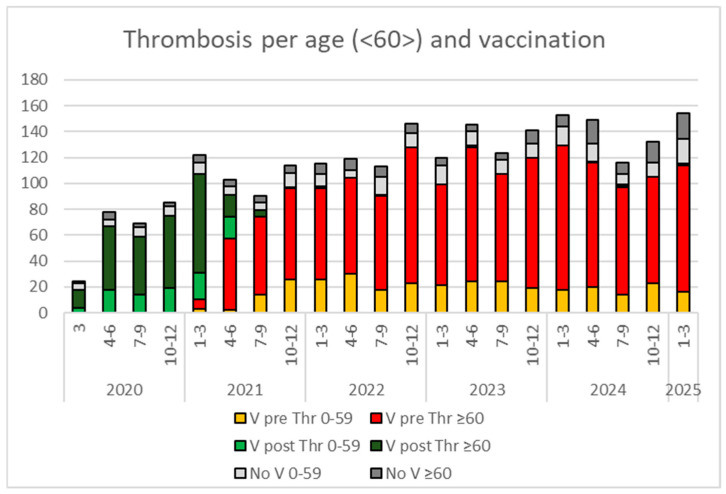
Total number of thrombotic events in relation to trimester (the specific number of months per year is indicated), age over or below 60 years old and vaccination prior (V pre Thr) or after (V post Thr) to the thrombosis, together with non- vaccinated patients (no V).

The relationship of thrombotic events with vaccination status and age (below or over 60 years old) is illustrated in Figure 3. Thrombotic events are rising in both vaccinated individuals and those without vaccination records, in people over and under 60 years old.

**Figure 4:**
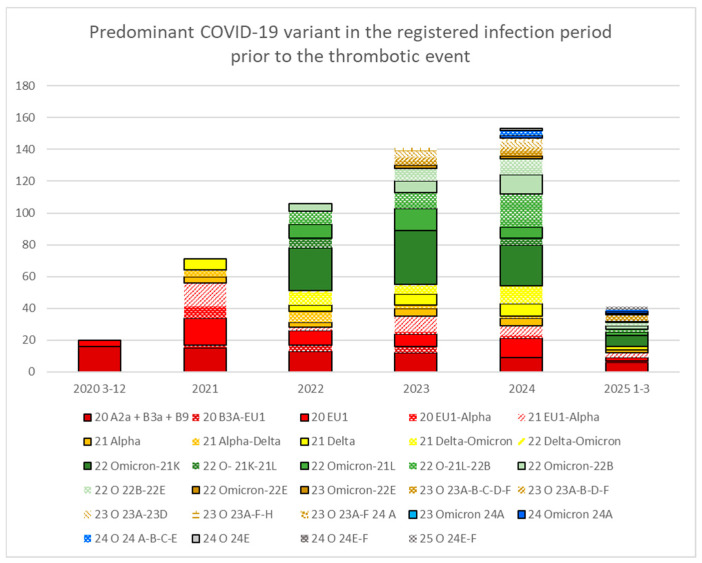
Predominant SARS-CoV-2 variant in the infection period prior to the thrombosis. The first number in each label indicates the year in which the variant appeared. The first two waves (in reddish tones) correspond to specific Spanish variants, while Alpha (orange) emerged at the end of 2020. Transitional periods between variants, before the next one becomes predominant, are shown as dotted, borderless columns. The Delta variant is shown in yellow, while the various Omicron variants appear in a green scale (2022), brown (2023), or blue-gray (2024). Note the reduced number of detected infections for variants that appeared after the end of full suspicion-based diagnoses in March 2022 (after Omicron 21K, which was predominant in the first trimester of 2022).

The predominant SARS-CoV-2 variant at the time of the first infection in patients who had at least one documented infection before the thrombosis is represented in Figure 4.

### 3.3. Thrombotic Events and Antihistamines

Overall, thrombotic events increased by 28% between 2021 and 2024, affecting 1.6% of AntiHm patients and 3.3% of No AntiHm patients with at least one chronic prescription (*p* < 0.0001 or <0.001 after a Benjamini-Hochberg correction) (Table 2). Thrombosis affected about a 4% of the population over 60 years old in most CST centers (File S4).

Significant ORs (1.52–2.53) were found (*) between the No AntiHm and AntiHm groups with at least 1 nt, when comparing thrombosis in vaccinated patients before or after the first infection and in non-infected patients (Table 3). Statistical differences that persist significant after a Benjamini–Hochberg correction are marked as ‘+’. Detailed data for age and nt subgroups, related to vaccination status and SARS-CoV-2 infection, are presented in File S2. No thrombosis appeared in patients treated incidentally with antihistamines who had no other chronic treatments (AntiHm 0 nT, File S2).

Logistic regression confirmed a significant and direct association of age, gender, and number of treatments with thrombosis (*p* < 0.001), and a significant inverse association with antihistamine treatment (*p* < 0.001) (File S3).

## 4. Discussion

To our knowledge, this is the first study to report an absence of long COVID in most subgroups of patients on chronic antihistamine therapy, alongside a substantial reduction in post-COVID-19 cardiovascular events in this population.

The present findings support the design of future prospective trials to confirm these observations, which should aim to include patients across all polypharmacy groups.

### 4.1. Antihistamines and Long COVID

Since an active medical code of Long COVID is suggestive of persistent symptoms, all active records have been grouped together in the tables. However, the limited number of long COVID patients in each AntiHm subgroup precludes definitive conclusions regarding the role of antihistamines in preventing long COVID. Chi-square tests require at least 5 cases in each subgroup, while multivariant regression analysis require at least 10 [24] while no statistics may be calculated when 0 cases appeared, which was the case in many categories. Further research is needed to elucidate if the absence of cases reflects a protective effect of Antihistamines and Long COVID.

Prior reports suggest a beneficial effect: no long COVID cases were documented following the pragmatic, empirical use of H1 antihistamines in primary care patients [16]. Furthermore, a randomized controlled trial of the H2 antihistamine famotidine reported improvement in cognitive and behavioral dysfunction following SARS-CoV-2 infection [25]. Other authors proposed histamine class-effect on neurological symptoms, mediated by several underlying mechanisms—including neuroactive ligand–receptor interactions. For instance, the antihistamine loratadine has been suggested to act as an antagonist of GRIN2B via this pathway [26]. GRIN genes encode N-methyl-D-aspartate receptors (NMDARs), and their dysregulation is linked to intellectual disability disorders [27].

### 4.2. Antihistamines, Respiratory Viruses and Thrombosis

While an increasing trend in thrombotic events was observed across all polypharmacy subgroups and centers in the non–AntiHm group (ranging from patients with no prior chronic conditions to those receiving ≥8 chronic treatments [nT]), a highly significant reduction was observed among AntiH-treated patients in most polypharmacy subgroups, suggesting a potential protective role, as previously reported for H1 antihistamines with respect to hospital admissions and mortality [14,16,17]. This antithrombotic effect is consistent with the previously reported effect of both H1 and H2 antihistamines on platelet-activating factor (PAF) [28]. The antihistamine H1 rupatadine is a well-known PAF antagonist [29]. Famotidine, an H2 antihistamine, was found to improve the rate of symptom resolution and reduce interferon-alpha plasma levels [30].

Other respiratory viruses, such as influenza, have been linked to thrombotic events [31,32,33]. While several respiratory viruses typically circulate concurrently each winter in the studied area, surveillance data from public programs [34] indicate that the winter of 2020–2021 had a surprisingly low incidence of influenza. Influenza was detected in only 0.4% of 256 positive samples collected by sentinel healthcare workers from randomly selected patients, compared to 18.4% for SARS-CoV-2 and 80.9% for other viruses (Table 2, [35]). Therefore, the increase in thrombosis observed in the first half of 2021 cannot be attributed to seasonal influenza infection.

Exposure to respiratory viruses with thrombotic potential, which circulate more commonly in winter, may explain the higher number of thrombotic events during that season. Previous research has reported a higher incidence of thrombotic thrombocytopenic purpura [36], myocardial infarction [37], and strokes in winter, the latter also being linked to increased pollution on colder days [38]. Our results, which show a greater number of thrombotic events during the colder months from October to March, are consistent with these data.

The higher proportion of thrombotic events in men is consistent with previous studies, since it is known that estrogen signaling is anti-inflammatory with vascular protective effects [39].

While the relationship between thrombosis and vaccination has yielded controversial results, with some studies reporting more events [40,41] and others dismissing an association [42], it is important to note that an increase in thrombosis was also observed in patients with no record of either vaccination or prior infection, as previously described [17]. Since COVID-19 has been reported to be thrombogenic through diverse mechanisms [43,44], the rise in thrombosis could be related to undiagnosed infections after 2022, which may be more frequent in the non-vaccinated group.

### 4.3. Limitations of the Study

The apparent rise in thrombotic events, assessed retrospectively in the population surviving as of 2025, could underestimate the true incidence by excluding patients who suffered early fatal thrombosis. However, the increase observed even among patients with low polypharmacy—who likely had higher survival rates—suggests that the rise is genuine.

The 15% increase in the population of the area during the last four years could also explain a global rise in thrombotic events, particularly in the subgroup without vaccination records, since they may represent patients who were not living in the CST area during the early stage of the pandemic. On the other hand, individuals below 60 years old had the possibility of being vaccinated outside the institution, and the rate of under-registration may be higher among those who, after four years, now belong to the group over 60. However, the rise observed in all polypharmacy subgroups, which implies the existence of vaccination records from early 2021, also suggests a long-lasting and real increase in thrombotic rates.

The presence of another private hospital in the area may have led some patients to seek care and receive diagnoses at that facility. Consequently, the number of events detected in our study could be an underestimate. However, the recording of chronic prescriptions is unlikely to be substantially affected by the use of private healthcare services, since patients generally return to the public health system to obtain subsidized long-term medications, and thrombotic events are subsequently recorded in their clinical records.

Furthermore, a chronic antihistamine prescription does not guarantee consistent medication intake. Nevertheless, no thrombosis cases were observed in patients with only occasional antihistamine prescriptions. Additionally, the prevalent use of pill organizers (containing the full prescribed allocation) among patients with higher polypharmacy suggests daily adherence. Future randomized prospective trials with a specific ‘intention-to-treat’ design are needed to confirm these findings.

## 5. Conclusions

Our results suggest an association between antihistamine use and reduced rates of thrombotic events. This protective effect requires confirmation through multicenter, randomized, prospective trials. However, this finding builds upon the previously established association between chronic antihistamine use and reduced hospital admissions and mortality. Furthermore, the reduction in thrombotic events was observed here across all polypharmacy groups. Together, this evidence supports considering antihistamines as a treatment option for symptomatic patients in the early stages of respiratory viral infections.

## Figures and Tables

**Table 1:** Analysis of Long COVID (LC) in relation to vaccination status and number of SARS-CoV-2 infections (n inf) among patients on chronic medication (nT > 1). The Odds ratio (OR) and confidence intervals (CI) of 95% for the percentage of patients presenting with Long COVID (%LC) are shown for parameters in the same row, comparing groups receiving antihistamine treatment (AntiHm) or not (No AntiHm), stratified by n inf and by whether vaccination was received before (V pre-inf) or after (V post-inf) the first infection. A more detailed analysis, stratified by increasing number of chronic medications, is provided in File S1.

		No AntiHm			AntiHm			OR %LC No AntiHm/ /AntiHm (CI 95%)	*p*
V	n inf	inf no LC	LC	%LC/ /(LC + inf no LC)	inf no LC	LC	%LC/ /(LC + inf no LC)		
	**1**								
V pre-inf		9806	51	0.5%	1526	12	0.8%	0.66 (0.35–1.24)	0.09
V post-inf		3478	94	2.7%	556	13	2.3%	1.16 (0.64–2.08)	0.31
No V		4786	42	0.9%	794	6	0.8%	1.16 (0.49–2.75)	0.36
	**2**								
V pre-inf		839	7	0.8%	152	0	0.0%		
V post-inf		808	34	4.2%	158	9	5.7%	0.74 (0.35–1.57)	0.21
No V		485	8	1.6%	115	2	1.7%	0.95 (0.20–4.53)	0.48
	**≥** **3**								
V pre-inf		77	2	2.6%	18	0	0.0%		
V post-inf		144	9	6.3%	22	0	0.0%		
No V		49	5	10.2%	10	0	0.0%		

**Table 2:** Absolute number of thrombotic events from 1 March 2020 to 28 March 2025, stratified by the number of chronic treatments (nT) in patients receiving acute antihistamine treatment without chronic therapies (AntiHm, nT = 0) or those on chronic antihistamine regimens (AntiHm) versus those not on chronic antihistamines (NoAntiHm). Odds ratios (ORs) are detailed for calendar years with complete data and for subgroups with more than five events. Asterisks (*) indicate ratios that were statistically significant in both one- and two-tailed comparisons. ‘+’ Indicates significance after a Benjamini–Hochberg correction.

Thrombosis	2020 3-12	2021	2022	2023	2024	2025 1-3	No Thrombosis	Total General
AntiHm/nT	13	26	38	34	38	13	10,253	10,415
0							1639	1639
1		1			1		2005	2007
2–4	3	9	6	2	10	1	3878	3909
5–7	1	4	14	14	13	5	1629	1680
≥8	9	12	18	18	14	7	1102	1180
No AntiHm/nT	243	403	455	495	512	141	179,987	182,236
0	18	31	23	21	41	28	117,980	118,142
1	8	9	17	22	33	9	19,556	19,654
2–4	48	88	111	120	159	40	25,608	26,174
5–7	83	117	151	165	145	30	10,639	11,330
≥8	86	158	153	167	134	34	6204	6936
No AntiHm ≥ 1 nT	225	372	432	474	471	113	62,007	64,094
Total general	256	429	493	529	550	154	190,240	192,651
**OR No AntiHm/AntiHm**								
2–4		1.5	2.8 *	9.0 *+	2.4 *+			
5–7		4.3 *+	1.6 *	1.7 *	1.7 *			
≥8		2.2 *+	1.4 *	1.6 *	1.6 *			
***p***								
2–4		0.13	0.005 *	0.0001 *	0.003 *			
5–7		0.0009 *	0.03 *	0.016 *	0.03 *			
≥8		0.002 *	0.049 *	0.02 *	0.03 *			
Confidence intervals (95%)								
2–4		(0.75–2.94)	(1.22–6.32)	(2.23–36.63)	(1.27–4.57)			
5–7		(1.62–11.93)	(0.94–2.82)	(1.03–3.10)	(0.97–3.02)			
≥8		(1.26–4.11)	(0.90–2.42)	1.00–2.66)	(0.97–2.95)			

**Table 3:** Number of thrombotic events in patients receiving at least one chronic treatment (≥1 nT), categorized by antihistamine use (AntiHm or NoAntiHm groups). For clarity, statistically significant results (*) are shown only for the comparison of patients who were both infected and vaccinated prior to the thrombotic event. ‘+’ Indicates significance after a Benjamini–Hochberg correction.

	No AntiHm ≥1 nT			AntiHm ≥1 nT			OR (*p*) (CI) No AntiHm/Antihm
	**V pre thr**	V post thr	No thr	**V pre thr**	V post thr	No Thr	
**V**	1470	324	43,834	123	21	5923	
**V preinf**	343	63	10,376	39	7	1662	
CoV							
**CoV pre Thr**	**260**	1		**27**			1.54 (1.03–2.30) (*p* = 0.01) *
CoV post thr	83	62		12	7		
CoV No Thr			**10,376**			**1662**	
**V postinf**	100	60	4407	9	6	743	
CoV							
**CoV pre Thr**	**100**	41		**9**	5		1.87 (0.94–3.72) (*p* = 0.03) *
CoV post thr		19			1		
CoV No Thr			**4407**			**743**	
**No CoV**	**1027**	201	**29,051**	75	**8**	**3518**	1.62 (1.30–2.09) (*p* = 0.00001) *+
**No V**			**Trh**-18466			**Thr**-2709	
CoV							
**CoV pre Thr**			**52**			**5**	1.81 (0.71–4.53) (*p* = 0.10)
CoV post thr			20			1	
CoV No Thr			**5303**			**921**	
No CoV							
**No Cov Thr**			**221**			**12**	2.53 (1.41–4.53) (*p* = 0.0006) *+
No CoV no Thr			**12,870**			**1770**	

## Data Availability

The original contributions presented in this study are included in the article. Further inquiries can be directed to the corresponding author.

## Supplementary Materials

The following supporting information can be downloaded at: https://www.mdpi.com/article/10.3390/v18020197/s1, File S0. Baseline characteristics. File S1. Percentage of long COVID depending on the number of infections (1, 2 or ≥3), the number of chronic treatments (0,1, 2–6 or ≥8), vaccination status, prior or after the first infection (V preinf or V postinf) or not vaccinated (No V), in patients receiving Antihistamine treatment (AntiHm) or not (No AntiHm). File S2. Number of thrombotic events depending on the number of chronic treatments (nt 0, 1 or ≥2), SARS-CoV-2 infection (‘CoV’ or ‘No inf’) prior or after the thrombosis (‘CoV pre Thr’ or ‘CoV post Thr’), together with vaccination status (‘V’ or ‘No V’), prior or after the first infection (‘V preinf’ or ‘V postinf’). File S3. Logistic regression analysis. File S4. Thrombotic events per center.

## Abbreviations

The following abbreviations are used in this manuscript:

AntiHm	Antihistamines
LC	Long COVID
No V	Non-vaccinated
Preinf	Previous to the SARS-CoV-2 infection
Postinf	Posterior to the SARS-CoV-2 infection
Pre Thr	Previous to the thrombosis
Post Thr	Posterior to the thrombosis
CoV	SARS-CoV-2 infection
CST	Consorci Sanitari de Terrassa
