# Supplementary material for: Long COVID and Reduced Thrombosis in Antihistamine-Treated Patients: An Observational Study in the Metropolitan Area of Barcelona
Source: Viruses. 2026 Feb 2;18(2):197. doi: 10.3390/v18020197 (PMC12945107; doi:10.3390/v18020197)
Supplement: Supplementary file 1 [file viruses-18-00197-s001.zip › Supplementary File 0_Baseline Characteristics.pdf]

Gender/	Infection/	n inf				nT		n V		Total general
/Age	/ Hospital admissions	1	2	>=3	no inf	Mean	±	Mean	±	
Women		26871	3245	404	66478	3.6	3.0	3.3	2.1	96998
1-19		3944	128	3	15734	1.4	0.9	1.8	0.6	19809
	Total infected	3944	128	3		1.5	1.0	1.6	0.7	4075
	Hospital admission	11		1		3.0	0.0	1.5	0.7	12
	No infection records				15734	1.4	0.9	1.9	0.6	15734
20-39		6830	984	118	15102	1.7	1.2	2.3	1.3	23034
	Total infected	6830	984	118		1.7	1.3	2.2	1.3	7932
	Hospital admission	52	15	3		1.9	1.1	1.5	0.7	70
	No infection records				15102	1.6	1.1	2.4	1.2	15102
40-59		9838	1449	178	18474	2.6	2.1	2.8	1.8	29939
	Total infected	9838	1449	178		2.6	2.1	2.7	1.7	11465
	Hospital admission	181	56	16		3.6	2.7	2.9	2.2	253
	No infection records				18474	2.6	2.0	2.9	1.9	18474
60-79		4793	537	80	12956	4.4	3.0	4.6	2.4	18366
	Total infected	4793	537	80		4.6	3.1	4.4	2.3	5410
	Hospital admission	295	68	25		5.9	3.6	4.4	2.6	388
	No infection records				12956	4.3	2.9	4.6	2.4	12956
>80		1466	147	25	4212	6.6	3.4	5.0	1.6	5850
	Total infected	1466	147	25		6.9	3.5	5.0	1.5	1638
	Hospital admission	179	38	16		7.8	3.9	4.9	1.6	233
	No infection records				4212	6.4	3.3	5.0	1.6	4212
Men		23315	1946	155	70237	3.5	2.9	3.3	2.3	95653
1-19		4107	124	2	16609	1.5	1.0	1.8	0.6	20842
	Total infected	4107	124	2		1.6	1.1	1.6	0.7	4233
	Hospital admission	16	1					1.8	1.0	17
	No infection records				16609	1.4	0.9	1.9	0.6	16609
20-39		6130	612	35	17425	1.8	1.3	2.2	1.1	24202
	Total infected	6130	612	35		1.8	1.4	2.0	1.2	6777
	Hospital admission	70	9	1		2.4	2.2	1.8	1.4	80
	No infection records				17425	1.7	1.3	2.3	1.1	17425
40-59		8502	790	73	21729	2.6	2.1	2.8	1.9	31094
	Total infected	8502	790	73		2.6	2.1	2.6	1.8	9365
	Hospital admission	284	52	14		2.9	2.2	2.7	2.1	350
	No infection records				21729	2.6	2.1	3.0	1.9	21729
60-79		3740	332	39	12104	4.3	3.0	4.8	2.6	16215
	Total infected	3740	332	39		4.5	3.1	4.6	2.6	4111
	Hospital admission	338	67	22		5.5	3.4	4.4	2.4	427
	No infection records				12104	4.3	2.9	4.8	2.6	12104
>80		836	88	6	2370	6.3	3.3	5.2	1.6	3300
	Total infected	836	88	6		7.1	3.5	5.2	1.4	930
	Hospital admission	157	34	3		7.8	3.8	5.0	1.4	194
	No infection records				2370	6.0	3.2	5.2	1.6	2370
Total general		50186	5191	559	136715	3.6	2.9	3.3	2.2	192651

S0a. Baseline characteristics of the total population of Consorci Sanitari de Terrassa (March 2025). including gender. age groups. number of infections (n inf). hospital admissions. and the mean ± standard deviation for the number of treatments (nT) and COVID-19 vaccines received (nV). A summary of the published results under a CC-BY license in the *Vaccines* journal on Thrombosis and Long COVID of the same population is reproduced below [6].

S0b- Previous data about centers demography to assess the role of antihistamines in reducing hospital admissions and deaths was published in [17] under a CC-BY license and are reproduced below.

The data of the healthcare area assigned to the CST, related to the three ABS with higher and lower life expectancy in Catalonia, are detailed in Table 1.

Table 1. Socioeconomic characteristics of the CST population and rate of infection. The basic healthcare area (ABS) in Terrassa and Rubí, belonging to the CST institution where this study was performed, are compared with the other six area in Catalonia with lower and higher life expectancy, together with the percentages of the population with lower and high incomes and incomplete primary education. The rate of infection in the assigned population is indicated in parenthesis beside the number of the assigned population. All areas showed vaccination rates of over 90% in the population of people over 60 years old treated with at least two chronic treatments.

Basic Healthcare Area (ABS)	Population 2024 (COV Infection)	Life Expectancy (2015)	Incomes <18,000 €	Incomes >100,000 €	Incomplete Primary Education	VAC in >60 + $\geq$2 nT
El Prat de Llobregat 3	12.996	79.0	66.8%	0.1%	32.6%	
St Adrià del Besós 2	17.579	79.2	71.5%	0.1%	34.5%	
St Quirze de Besora	4.834	79.7	72.6%	0.4%	20.1%	
CST ABS						
TERRASSA B	28,399 (24%)	81.7	69.6%	0.04%	26.6%	90.7%
RUBI 3	17,796 (21%)	82.0	63.1%	0.3%	22.3%	94.2%
TERRASSA F (+Matadapera)	37,164 (22 + 24%)	83.1	67.6%	1.4%	24.1%	93.3% (+92.5%)
RUBÍ 2 (+Castellbisbal)	41,100 (27 + 24%)	83.3	65.2%	0.3%	21.8%	92.7% (+93.3%)
TERRASSA A	22,972 (23%)	83.8	58.8%	1.1%	16.9%	92.3%
Sta Perpètua de Mogoda	25,821	86.7	62.2%	0.27%	25.1%	
Barcelona 8-C: Turó de la Peira	23,968	86.7	69.4%	0.2%	27.1%	
Sant Cugat del Vallès 2	33,911	87.0	45.0%	7.8%	5.8%	

S0c. Previous data about date of Long COVID diagnoses and thrombotic events and reduction of diagnoses after the end of full suspicion diagnosis were published in [6] under a CC-BY license and are reproduced below.

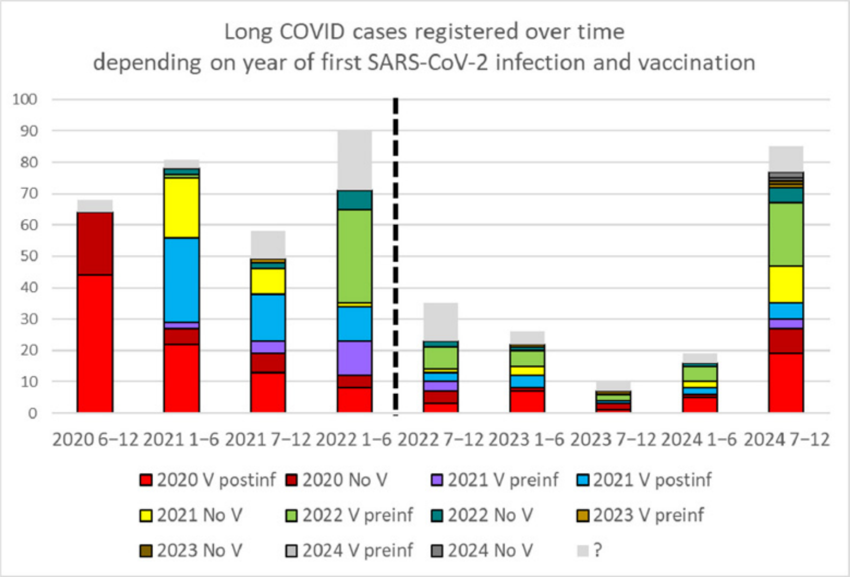

Figure 1. Long COVID cases detected every six months (1–6 indicate the first half of the year, and 7–12 the second half). The year of the first COVID-19 infection is also shown, indicating whether vaccination occurred prior to infection (V preinf), after infection (V postinf), or not at all (No V). The dashed line marks the end of the protocol recommending testing for any symptomatic patient. The increase observed in 2024 coincides with active case finding in the CST via survey. In some long COVID records, the exact date of infection is uncertain, likely due to previous unreported self-diagnosis by the patient. Those cases with uncertain data of infection are indicated as '?'.

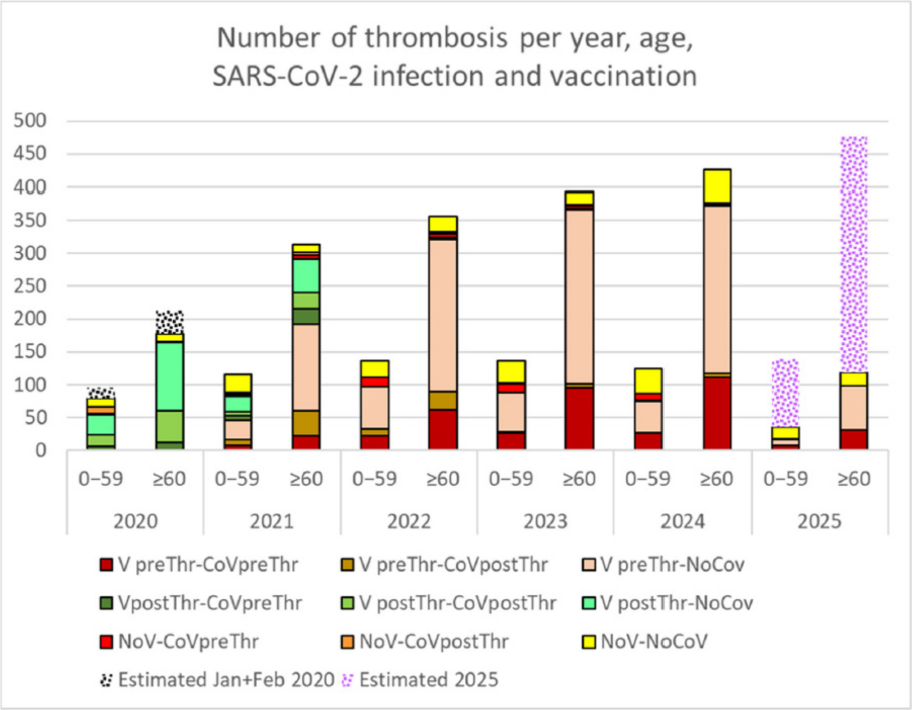

Figure 3. Cumulative thrombotic events from March 2020 to March 2025, stratified by age (≥60 vs. <60 years), vaccination status (vaccinated [V] vs. non-vaccinated [NoV]) and SARS-CoV-2 infection (CoV or NoCoV, together with the temporal relation to thrombotic events, either before (preThr) or after (postThr) the event). Annual thrombosis estimates (2020, 2025) were extrapolated from monthly averages of recorded events, adjusting for months with missing data.

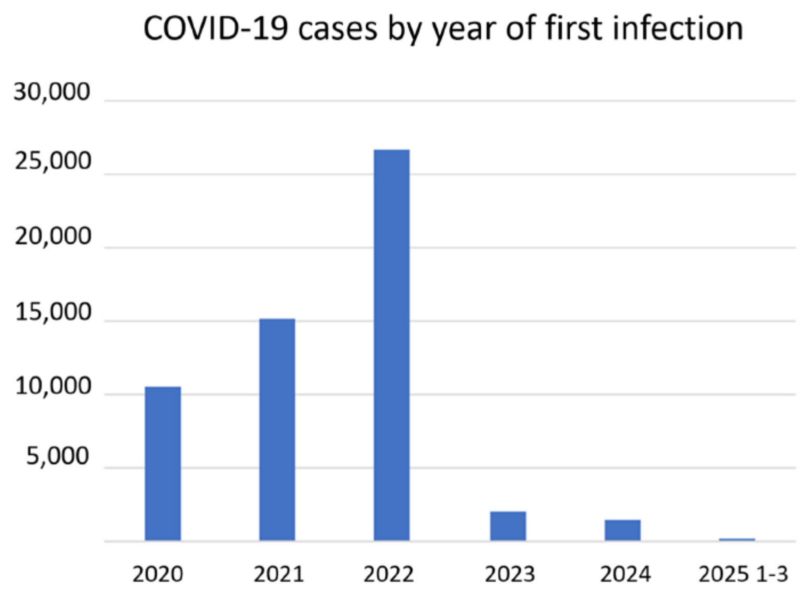

Figure 2. Period of the first SARS-CoV-2 infection. The number of detected cases decreased dramatically in March 2022, after the end of protocols that required case detection beyond symptomatic patients.
