# Supplementary material for: Long COVID and Reduced Thrombosis in Antihistamine-Treated Patients: An Observational Study in the Metropolitan Area of Barcelona
Source: Viruses. 2026 Feb 2;18(2):197. doi: 10.3390/v18020197 (PMC12945107; doi:10.3390/v18020197)
Supplement: Supplementary file 1 [file viruses-18-00197-s001.zip › Supplementary File 1_LC new.pdf]

n inf	1			2			≥3			0 or ?			Total general
Inf-LC-%	Inf no LC	LC	% LC	Inf no LC	LC	% LC	Inf no LC	LC	% LC	?Inf	LC	% LC	
**No AntiHm**	46481	276	0.6%	4624	64	1.4%	483	24	4.9%	130229	55	0.04%	182236
V preinf	18303	73	0.4%	1363	10	0.7%	126	2	1.6%				19877
0	8497	22	0.3%	524	3	0.6%	49		0.0%				9095
1	2410	12	0.5%	212		0.0%	18		0.0%				2652
2-7	5937	31	0.5%	496	6	1.2%	43	2	4.4%				6515
>8	1459	8	0.5%	131	1	0.8%	16		0.0%				1615
V postinf	7600	116	1.5%	1559	38	2.4%	232	14	6.0%				9559
0 nT	4122	22	0.5%	751	4	0.5%	88	5	5.4%				4992
1 nT	939	23	2.4%	240	9	3.6%	43		0.0%				1254
2-7 nT	2088	63	2.9%	464	23	4.7%	79	8	9.2%				2725
>8 nT	451	8	1.8%	104	2	1.9%	22	1	4.3%				588
No V	20578	87	0.4%	1702	16	0.9%	125	8	6.4%	130229	55	0.04%	152800
0 nT	15792	45	0.3%	1217	8	0.7%	76	3	3.8%	86897	17	0.02%	104055
1 nT	2427	19	0.8%	230	4	1.7%	27		0.0%	13032	9	0.07%	15748
2-7 nT	2266	23	1.0%	246	3	1.2%	21	5	19.2%	25676	24	0.09%	28264
>8 nT	93		0.0%	9	1	10.0%	1		0.0%	4624	5	0.11%	4733
**AntiHm**	3395	34	1.0%	492	11	2.2%	52		0.0%	6420	11	0.17%	10415
V preinf	1708	14	0.8%	170		0.0%	19		0.0%				1911
0 nT	182	2	1.1%	18		0.0%	1		0.0%				203
1 nT	291	1	0.3%	25		0.0%	2		0.0%				319
2-7 nT	978	6	0.6%	92		0.0%	15		0.0%				1091
>8 nT	257	5	1.9%	35		0.0%	1		0.0%				298
V postinf	657	13	1.9%	191	9	4.7%	22		0.0%				892
0 nT	101		0.0%	33		0.0%			0.0%				134
1 nT	121	3	2.5%	35	2	5.4%	5		0.0%				166
2-7 nT	339	7	2.0%	106	5	4.5%	15		0.0%				472
>8 nT	96	3	3.1%	17	2	10.5%	2		0.0%				120
No V	1030	7	0.7%	131	2	1.5%	11		0.0%	6420	11	0.17%	7612
0 nT	236	1	0.4%	16		0.0%	1		0.0%	1048		0.00%	1302
1 nT	253		0.0%	33		0.0%	3		0.0%	1231	2	0.16%	1522
2-7 nT	510	5	1.0%	74	2	2.6%	4		0.0%	3424	7	0.20%	4026
>8 nT	31	1	3.1%	8		0.0%	3		0.0%	717	2	0.28%	762
Total general	49876	310	0.6%	5116	75	1.5%	535	24	4.5%	136649	66	136715	192651

S1. Percentage of long COVID depending on the number of infections (1. 2 or ≥3), the number of chronic treatments (0.1. 2-6 or ≥8), vaccination status, prior or after the first infection (V preinf or V postinf) or not vaccinated (No V), in patients receiving Antihistamine treatment (AntiHm) or not (No AntiHm). Patients without chronic treatments who received occasional treatment with antihistamines are recorded as 0 nT in the AntiHm section.
