# Supplementary material for: Long COVID and Reduced Thrombosis in Antihistamine-Treated Patients: An Observational Study in the Metropolitan Area of Barcelona
Source: Viruses. 2026 Feb 2;18(2):197. doi: 10.3390/v18020197 (PMC12945107; doi:10.3390/v18020197)
Supplement: Supplementary file 1 [file viruses-18-00197-s001.zip › Supplementary File 2_ V x nt i edat i antithm.pdf]

Supplementary data S2. Number of thrombotic events depending on the number of chronic treatments (nt 0, 1 or >=2), SARS-CoV-2 infection ('Cov' or 'No inf') prior or after the thrombosis ('CoV pre Thr' or 'CoV post Thr'), together with vaccination status ('V' or 'No V'), prior or after the first infection ('V preinf' or 'V postinf').

	**V**									**No V**	
	**V preinf**			**V postinf**			**No inf**				
	**CoV pre Thr**	**CoV post Trh**	**CoV no Thr**	**CoV pre Thr**	**CoV post Trh**	**CoV no Thr**	**V pre Thr**	**V post Trh**	**V no Thr**		Total general
**No AntiHm/age/nT**	**356**	**70**	**19451**	**109**	**66**	**9384**	**1082**	**207**	**55692**	**95819**	**182236**
**0-59**	**63**	**11**	**12932**	**22**	**15**	**6806**	**159**	**35**	**32660**	**89135**	**141838**
0											
**CoV**											
Inf no Thr			8448			4646				16636	29730
CoV pre Thr	6			3	2					6	17
CoV post Thr		1			2					4	7
**No inf**											
No Inf no Trh									23517	57660	81233
No Inf Thr							24	2		30	56
1											
**CoV**											
Inf no Thr			1985			969				2487	5441
CoV pre Thr	7			2						1	10
CoV post Thr										1	1
**No inf**											
No inf no Thr									3840	5886	9726
No inf Thr							14			21	35
>=2											
**CoV**											
Inf no Thr			2499			1191				2012	5702
CoV pre Thr	39			17	6					29	91
CoV post Thr	11	10			5					7	33
**No inf**											
No inf no Thr									**5303**	**4272**	**9575**
No inf Thr							**121**	**33**		**83**	**237**
**>=60**	**293**	**59**	**6519**	**87**	**51**	**2578**	**923**	**172**	**23032**	**6684**	**40398**
0											
**CoV**											
Inf no Thr			627			331				493	1451
CoV pre Thr	6			6	1					2	15
CoV post Thr	1	6			1						8
**No inf**											
No inf no Thr									3124	2498	5622
No inf Thr							31	4		24	**59**
1											
**CoV**											
Inf no Thr			652			280				218	1150
CoV pre Thr	3			2	1						6
CoV post Thr	3	2									5
**No inf**											
No inf no Thr									2510	729	**3239**
No inf Thr							31	3		7	41
>=2											
**CoV**											
Inf no Thr			5240			1967				586	7793
CoV pre Thr	211	1		79	34					22	347
CoV post Thr	69	50			14					12	145
**No inf**											
No inf no Thr									17398	1983	19381
No inf Thr							861	165		110	1136

	V									No V	
	V preinf			V postinf			No inf				
	CoV pre Thr	CoV post Trh	CoV no Thr	CoV pre Thr	CoV post Trh	CoV no Thr	V pre Thr	V post Trh	V no Thr		Total general
**AntiHm**	**39**	**7**	**1865**	**9**	**6**	**877**	**75**	**8**	**3966**	**3563**	**10415**
**0-59**	**8**	**2**	**1130**	**2**		**593**	**16**	**1**	**2091**	**3239**	**7082**
0											
**CoV**			188			128				248	564
**No inf**									408	589	997
1											
**CoV**			269			141				280	690
**No inf**									471	625	1096
>=2											
**CoV**											
Inf no thr			673			324				550	1547
CoV pre Thr	4			2						2	8
CoV post Thr	4	2									6
**No inf**											
No inf no Thr									1212	938	2150
No inf Thr							16	1		7	24
**>=60**	**31**	**5**	**735**	**7**	**6**	**284**	**59**	**7**	**1875**	**324**	**3333**
0											
**CoV**			15			6				6	27
**No inf**									40	11	51
1											
**CoV**											
Inf no Thr			49			25				9	83
CoV pre Thr	1										1
**No inf**											
No inf no Thr									103	33	136
No inf Thr							1				1
>=2											
**CoV**											
Inf no Trh			671			253				82	1006
CoV pre Thr	22			7	5					3	37
CoV post Thr	8	5			1					1	15
**No inf**											
No inf no Thr									1732	174	1906
No inf Thr							58	7		5	70
**Total general**	**395**	**77**	**21316**	**118**	**72**	**10261**	**1157**	**215**	**59658**	**99382**	**192651**
