# Supplementary material for: Long COVID and Reduced Thrombosis in Antihistamine-Treated Patients: An Observational Study in the Metropolitan Area of Barcelona
Source: Viruses. 2026 Feb 2;18(2):197. doi: 10.3390/v18020197 (PMC12945107; doi:10.3390/v18020197)
Supplement: Supplementary file 1 [file viruses-18-00197-s001.zip › Supplementary File 3_logistic regression THR and LC.pdf]

S3. Classical and Bayesian logistic regression analyses, including multicollinearity diagnostics, were conducted for thrombosis and Long COVID. A direct and statistically significant association with thrombosis was identified for age, sex, and the number of chronic treatments (nT), whereas an inverse association, indicating a reduction in events, was observed for antihistamine use (antiHm). Although logistic regression models were computed for Long COVID, the minimum requirement of 10 cases per subgroup was not met; therefore, these results cannot be considered conclusive.

Logistic Regression ▾

Model Summary - Thrombus

Model	Deviance	AIC	BIC	df	ΔX²	p	McFadden R²	Nagelkerke R²	Tjur R²
M_0	25916	25918.104	25928.273	192650			0.000		0.000
M_1	7253	7267.264	7338.444	192644	18662.840	< .001	0.720	0.734	0.741

Note. M_1 includes Age, Gender, nT, VCoV preThr, AntiHm, COV preThr

Coefficients

Model		Estimate	Standard Error	Odds Ratio	z	Wald Test: Wald Statistic	Wald Test: df	Wald Test: p
M_0	(Intercept)	−4.368	0.020	0.013	−213.143	45429.988	1	< .001
M_1	(Intercept)	−8.489	0.163	2.058×10^{-4}	−52.085	2712.799	1	< .001
	Age	0.033	0.003	1.034	12.739	162.271	1	< .001
	Gender (H)	0.814	0.084	2.257	9.655	93.219	1	< .001
	nT	0.197	0.011	1.217	17.950	322.192	1	< .001
	VCoV preThr (1)	23.736	217.103	$2.034\times10^{+10}$	0.109	0.012	1	.913
	AntiHm (S)	−0.713	0.193	0.490	−3.687	13.591	1	< .001
	COV preThr (1)	22.885	347.603	$8.683\times10^{+9}$	0.066	0.004	1	.948

Note. Thrombus level '1' coded as class 1.

Multicollinearity Diagnostics ▾

	Tolerance	VIF
Age	0.631	1.584
Gender	0.980	1.020
nT	0.626	1.598
VCoV preThr	1.000	1.000
AntiHm	0.982	1.018
COV preThr	1.000	1.000

Bayesian Logistic Regression ▾

Model Comparison - Thrombus ▾

Models	P(M)	P(M|data)	BF_M	BF_{10}	R²
Age	0.083	0.977	458.589	1.000	0.165
AntiHm + Age	0.083	0.023	0.264	0.024	0.165
Age + nT	0.083	9.197×10^{-63}	1.012×10^{-61}	9.418×10^{-63}	0.208
AntiHm + Age + nT	0.250	1.717×10^{-70}	5.152×10^{-70}	5.862×10^{-71}	0.210
Null model	0.250	0.000	0.000	0.000	0.000
AntiHm + nT	0.083	0.000	0.000	0.000	0.172
nT	0.083	0.000	0.000	0.000	0.170
AntiHm	0.083	0.000	0.000	0.000	0.000

Results ▼

Logistic Regression ▼

Model Summary - Long Covid

Model	Deviance	AIC	BIC	df	ΔΧ²	p	McFadden R²	Nagelkerke R²	Tjur R²	Cox & Snell R²
M_0	4838	4840.129	4849.061	55935			0.000		0.000	
M_1	4585	4598.873	4661.397	55929	253.256	< .001	0.052	0.055	0.008	0.005

Note. M_1 includes ncov, age, gender, nT, VCoVpreinf, AntiHm

Coefficients ▼

Model		Estimate	Standard Error	Odds Ratio	z	Wald Test		
						Wald Statistic	df	p
M_0	(Intercept)	−4.911	0.050	0.007	−98.962	9793.398	1	< .001
M_1	(Intercept)	−6.175	0.180	0.002	−34.372	1181.434	1	< .001
	ncov	0.661	0.074	1.936	8.965	80.378	1	< .001
	age	0.017	0.003	1.018	5.428	29.465	1	< .001
	gender (H)	−0.490	0.107	0.612	−4.577	20.950	1	< .001
	nT	0.065	0.018	1.068	3.539	12.524	1	< .001
	VCoVpreinf (1)	−1.008	0.122	0.365	−8.264	68.294	1	< .001
	AntiHm (S)	0.234	0.164	1.263	1.427	2.036	1	.154

Note. Long Covid level 'S' coded as class 1.

Bayesian Logistic Regression

Model Comparison - Long Covid

Models	P(M)	P(M|data)	BF_M	BF_{10}	R²
gender + VCoVpreinf + ncov + age + nT	9.440×10^{-4}	0.260	372.646	1.000	0.052
gender + VCoVpreinf + gender:VCoVpreinf + ncov + age + nT	0.001	0.161	169.574	0.516	0.052
gender + VCoVpreinf + AntiHm + ncov + age + nT	0.001	0.107	105.296	0.341	0.052
gender + VCoVpreinf + gender:VCoVpreinf + AntiHm + ncov + age + nT	0.002	0.106	59.916	0.194	0.053
gender + VCoVpreinf + gender:VCoVpreinf + AntiHm + gender:AntiHm + VCoVpreinf:AntiHm + gender:VCoVpreinf:AntiHm + ncov + age + nT	0.238	0.095	0.334	0.001	0.053
gender + VCoVpreinf + gender:VCoVpreinf + AntiHm + gender:AntiHm + ncov + age + nT	0.005	0.067	13.577	0.046	0.053
gender + VCoVpreinf + gender:VCoVpreinf + AntiHm + gender:AntiHm + VCoVpreinf:AntiHm + ncov + age + nT	0.024	0.052	2.271	0.008	0.053
gender + VCoVpreinf + gender:VCoVpreinf + AntiHm + VCoVpreinf:AntiHm + ncov + age + nT	0.005	0.047	9.332	0.032	0.053
gender + VCoVpreinf + AntiHm + gender:AntiHm + ncov + age + nT	0.002	0.044	22.905	0.080	0.053
gender + VCoVpreinf + AntiHm + VCoVpreinf:AntiHm + ncov + age + nT	0.002	0.029	15.301	0.054	0.052

Note. Table displays only a subset of models; to see all models, select "No" under "Limit No. Models Shown".
