# Supplementary material for: Long COVID and Reduced Thrombosis in Antihistamine-Treated Patients: An Observational Study in the Metropolitan Area of Barcelona
Source: Viruses. 2026 Feb 2;18(2):197. doi: 10.3390/v18020197 (PMC12945107; doi:10.3390/v18020197)
Supplement: Supplementary file 1 [file viruses-18-00197-s001.zip › Supplementary File 4_thrombosis per center.pdf]

	Thrombosis / year						Total number of thrombosis	No Thrombosis	Total general	% total thrombosis
	2020	2021	2022	2023	2024	2025				
**No AntiHm**										
0-59										
CAP MATADEPERA	1	1	3	8	2		15	7207	7222	0,21%
CAP DR JOAN PLANAS	6	1	3	10	10		30	8586	8616	0,35%
CAP SANT GENIS	3	9	9	13	9	3	46	12347	12393	0,37%
CAP CAN ROCA	3	4	14	15	7	7	50	15639	15689	0,32%
CAP TERRASSA EST	14	11	15	21	19	7	87	23028	23115	0,38%
CAP TERRASSA NORD	9	11	17	8	14	7	66	19918	19984	0,33%
CAP ANTON DE BORJA	5	16	20	24	17	5	87	22632	22719	0,38%
CAP SANT LLATZER	12	27	20	17	25	5	106	31994	32100	0,33%
≥60										
CAP MATADEPERA	12	11	15	16	30	5	89	2331	2420	3,68%
CAP DR JOAN PLANAS	8	19	12	16	37	11	103	2154	2257	4,56%
CAP SANT GENIS	19	22	37	34	32	6	150	3244	3394	4,42%
CAP CAN ROCA	18	27	50	48	42	9	194	4514	4708	4,12%
CAP TERRASSA EST	19	39	26	46	44	9	183	4079	4262	4,29%
CAP TERRASSA NORD	26	45	53	64	67	15	270	5896	6166	4,38%
CAP ANTON DE BORJA	34	63	69	65	77	16	324	6551	6875	4,71%
CAP SANT LLATZER	54	97	92	90	80	36	449	9867	10316	4,35%
**AntiHm**										
0-59										
CAP MATADEPERA		1					1	216	217	0,46%
CAP DR JOAN PLANAS		1	1	1	2		5	434	439	1,14%
CAP SANT GENIS								407	407	0,00%
CAP CAN ROCA		1	1	1	2		5	743	748	0,67%
CAP TERRASSA EST		1	2		2		5	1166	1171	0,43%
CAP TERRASSA NORD			1		1	1	3	944	947	0,32%
CAP ANTON DE BORJA		3	1	2	2		8	1411	1419	0,56%
CAP SANT LLATZER	1	1	4	1	4		11	1723	1734	0,63%
≥60										
CAP MATADEPERA	1	1	1	1	2		6	160	166	3,61%
CAP DR JOAN PLANAS		1	1	1	4		7	140	147	4,76%
CAP SANT GENIS		1	2	2	2	1	8	203	211	3,79%
CAP CAN ROCA		1	3	4	3	3	14	323	337	4,15%
CAP TERRASSA EST	2		2	3	1	4	12	390	402	2,99%
CAP TERRASSA NORD	5	4	5	3	3	1	21	455	476	4,41%
CAP ANTON DE BORJA	1	5	8	7	2	2	25	658	683	3,66%
CAP SANT LLATZER	3	5	6	8	8	1	31	880	911	3,40%
Total general	256	429	493	529	550	154	2411	190240	192651	1,25%

Supplementary data S4. Absolute number of thrombosis per year and center, in population over or below 60 years old. The % of thrombosis in the older group is above 4% in all centers excepting the residential area of Matadapera (where reside less than 5% of the population of patients of more than 60 years of the institution). Probably, residents affected by thrombosis have moved to other more urban area.
